# The MEK5/ERK5 pathway promotes the activation of the Hedgehog/GLI signaling in melanoma cells

**DOI:** 10.1007/s13402-025-01050-z

**Published:** 2025-02-25

**Authors:** Ignazia Tusa, Sinforosa Gagliardi, Alessio Menconi, Luisa Maresca, Alessandro Tubita, Matteo Lulli, Barbara Stecca, Elisabetta Rovida

**Affiliations:** 1 Department of Experimental and Clinical Biomedical Sciences “Mario Serio”, University of Florence, 50134 Florence, Italy; 2 Core Research Laboratory - Institute for Cancer Research and Prevention (ISPRO), Florence, Italy

**Keywords:** ERK5/*MAPK7*, Hedgehog, GLI, Targeted therapy, Melanoma

## Abstract

**Purpose:**

Malignant melanoma is the deadliest skin cancer, with a poor prognosis in advanced stages. We reported that both Hedgehog-GLI (HH/GLI) and Mitogen-activated protein Kinase (MAPK) extracellular signal-regulated kinase 5 (ERK5) pathways promote melanoma growth, and that ERK5 activation is required for HH/GLI-dependent melanoma cell proliferation. Here, we explored whether ERK5 regulates HH/GLI signaling.

**Methods:**

Both genetic (using ERK5-specific shRNA) and pharmacologic (using the ERK5 inhibitors JWG-071 and AX15836, and the MAPK/ERK kinase 5, MEK5 inhibitors GW284543 and BIX02189) targeting approaches were used. Luciferase assay using the GLI-binding site luciferase reporter was performed to evaluate GLI transcriptional activity. A constitutively active form of MEK5 (MEK5DD) was used to induce ERK5 activation. 3D spheroid assays were performed in melanoma cells.

**Results:**

Genetic and pharmacologic ERK5 inhibition reduces GLI1 and GLI2 protein levels and transcriptional activity of endogenous HH/GLI pathway induced by the agonist SAG in NIH/3T3 cells. In these cells, MEK5DD overexpression potentiates transcriptional activity of endogenous HH/GLI pathway induced by SAG, whereas ERK5 silencing prevents this effect. Consistently, MEK5DD overexpression increases GLI1 and GLI2 protein levels. In melanoma cells, ERK5 silencing reduces GLI1 and GLI2 mRNA and protein levels and inhibits GLI transcriptional activity. MEK5DD further increases the transcriptional activity of the HH/GLI pathway and GLI1 protein levels. Combination of GLI and MEK5 inhibitors is more effective than single treatments in reducing melanoma spheroid growth.

**Conclusions:**

MEK5-ERK5 is an activator of GLI transcription factors, and combined targeting of these pathways warrants further preclinical investigation as a potential innovative therapeutic approach for melanoma.

**Graphical abstract:**

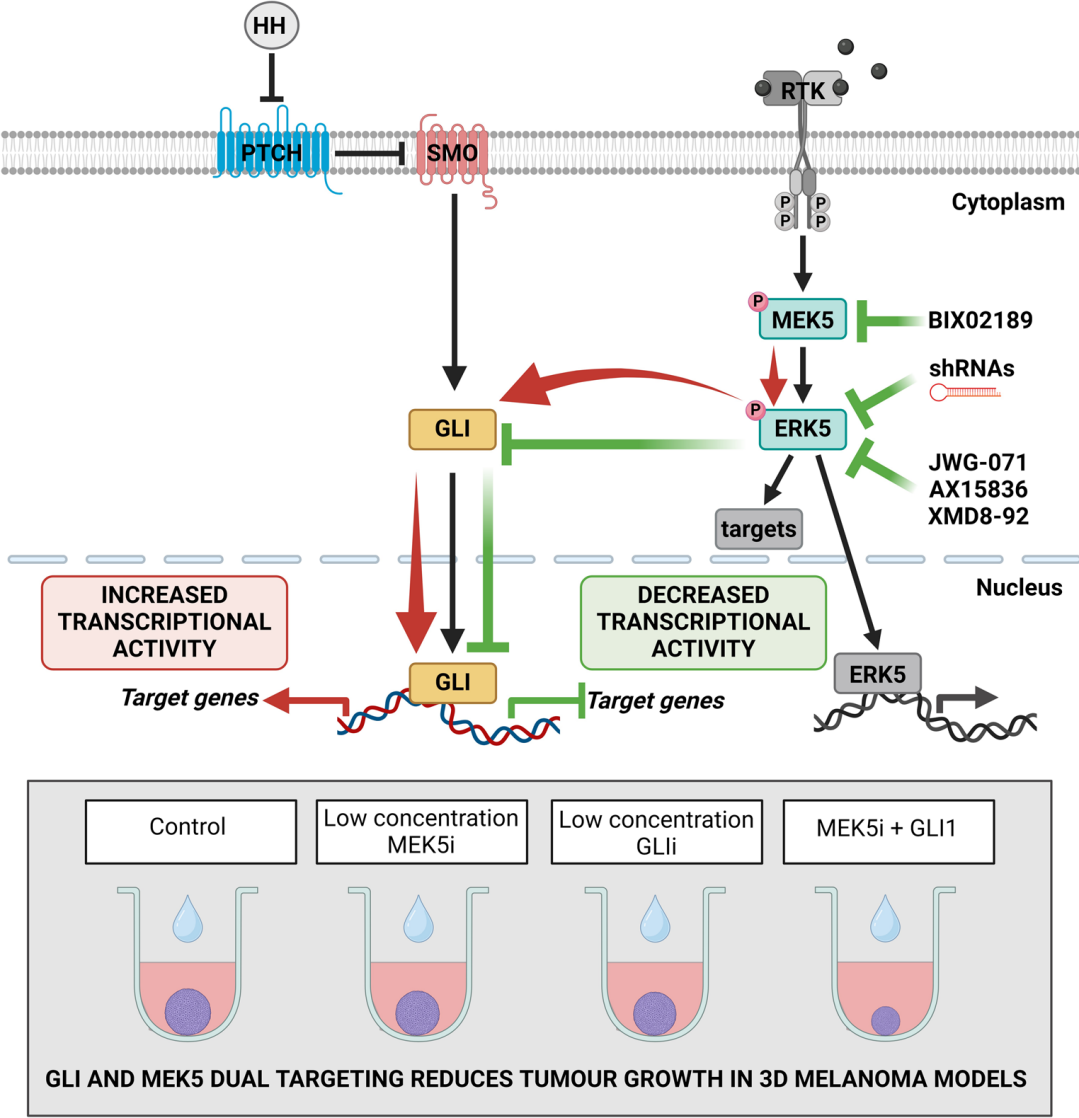

**Supplementary Information:**

The online version contains supplementary material available at 10.1007/s13402-025-01050-z.

## Introduction

The extracellular signal-related kinase 5 (ERK5), also referred to as big mitogen-activated kinase 1 (BMK1), belongs to the mitogen-activated protein kinase (MAPK) family [1]. ERK5 is involved in several biological responses, including cell survival, anti-apoptotic signaling, angiogenesis, differentiation and proliferation of several cell types [1], and plays a relevant role in the onset and progression of cancer [2]. ERK5 activation is achieved through MEK5-dependent or -independent phosphorylation that stimulates ERK5 nuclear translocation via importin beta 1 [3], a key event for cell proliferation [4, 5].

The Hedgehog/GLI (HH/GLI) signaling pathway plays a central role during embryonic development, and is involved in cell proliferation, differentiation and tissue patterning. In adults, HH/GLI signaling is rapidly turned off and remains active in stem cells where it maintains tissue homeostasis and regeneration [6]. There are two main types of HH/GLI signaling pathway activation: canonical and non-canonical signaling [7]. Canonical HH/GLI signaling is triggered by the binding of HH ligands to the 12-pass transmembrane receptor Patched 1 (PTCH1). With this binding, PTCH1 no longer represses the 7-pass transmembrane G protein-coupled receptor Smoothened (SMO), thus allowing the intracellular activation of the zinc finger transcription factors GLI2 and GLI3, which translocate into the nucleus and transactivate GLI1 promoter. In addition, several types of cancer, including melanoma, present non-canonical activation of the GLI transcription factors through multiple oncogenic inputs independent of upstream PTCH/SMO signaling [8]. Both mechanisms of HH/GLI pathway activation (canonical and non-canonical) lead to increased levels of GLI proteins, which enhance transcription of target genes and promote tumor growth and progression [8, 9]. Non-canonical HH/GLI signaling activation by the MAPK pathway has been described in normal as well as cancer cells [10]. We previously established that both HH/GLI and MEK5/ERK5 pathways are required for melanoma growth [11–13], and that ERK5 is required for HH/GLI-dependent melanoma cell proliferation [14]. This study aimed to evaluate whether the MEK5/ERK5 pathway is involved in the activation of the HH/GLI signaling and if this interplay may be exploited to reduce melanoma growth.

## Materials and methods

### Cell lines and treatments

Human melanoma cell lines A375 and SK-Mel-5, murine NIH/3T3 fibroblasts, human HEK-293T and HeLa cells were obtained from ATCC (Manassas, VA, U.S.A.). Patient-derived SSM2c melanoma cells have been already described [15, 16]. All cells were maintained in Dulbecco’s modified Eagle’s medium (DMEM) supplemented with 10% heat-inactivated foetal bovine serum (FBS), 2 mM glutamine, 50 U/mL penicillin, and 50 mg/mL streptomycin (Euroclone, Milan, Italy), and incubated at 37 °C in a water-saturated atmosphere containing 95% air (21% O_2_) and 5% CO_2_. Cell lines were authenticated by cell profiling (Promega PowerPlex Fusion System kit; BMR Genomics S.R.L., Padova, Italy) once a year. The mycoplasma contamination of cell cultures was periodically excluded by PCR. Cultures were renewed every 3 months.

ERK5 inhibitors JWG-071 [17], AX15836 [18] and XMD8-92 [19], MEK5 inhibitors GW284543 [20] and BIX02189 [21] (MedChemExpress LLC, Princeton, NJ, U.S.A.), the GLI1/2 inhibitor GANT61 [22] and the SMO agonist SAG [23] (Sigma-Aldrich, St Louis, MO, U.S.A. and MedChemExpress LLC, Princeton, NJ, U.S.A.) were dissolved in DMSO.

### Cell lysis and Western Blot

Total cell lysates were obtained using Laemmli or RIPA buffer, as previously described [24]. Nucleus-cytoplasm fractions have been obtained as previously described [25]. Proteins were separated by SDS-PAGE and transferred onto Hybond™ PVDF membranes (GE Healthcare, IL, U.S.A.) by electroblotting as previously described [26]. Infrared imaging (Odyssey, Li-Cor Bioscience, Lincoln, NE, U.S.A.) was used to detect protein bands. Images were recorded as TIFF files for quantification with ImageJ software. Antibodies used are listed in Supplementary Table S1.

### RNA interference

Lentiviruses were produced in HEK-293T cells as previously reported [27]. Lentiviral vectors for stable silencing of *erk5* in murine NIH/3T3 fibroblasts and in human melanoma cells were TRC1.5-pLKO.1-puro vector containing shRNA sequences (shNT), or target-specific sequences (Supplementary Table S2). Transduced cells were selected with 2 µg/ml puromycin for at least 72 h.

### Plasmids and transfection

The pCMV5-MEK5DD-HA vector encoding for a constitutively active form of MEK5 was generously provided by Jiing-Dwan Lee (Scripps Institute, La Jolla, CA, USA). pcDNA3.1 empty vector was used as control. Vectors used for overexpression were: pCS2 + MT (Addgene, Cambridge, MA, USA) and Myc-tagged human GLI1 (pCS2 + MT-GLI1, kind gift from A. Ruiz i Altaba) [28]. SSM2c cells were plated on six-well dishes (3 × 10^5^ cells/well) and transfected after 24 h with a total amount of 2 µg of plasmid DNA usingLipofectamine 2000 (Invitrogen, Thermo Fisher Scientific), following manufacturer’s instructions. Cells were lysed after 24–48 h.

### Luciferase assay

The GLI-responsive luciferase reporter (8 × 3’GLI-BS) [29] was used in combination with *Renilla* luciferase pRL-TK reporter vector (Promega Corporation, Madison, WI, U.S.A.) to normalize luciferase activities; pGL3Basic vector (Promega Corporation) was used to equal DNA amounts as previously reported [30]. Luminescence was measured using the Dual-Glo Luciferase Assay System (Promega Corporation) and the GloMax 20/20 Luminometer (Promega Corporation).

### Quantitative real-time (Q-PCR)

Total RNA was isolated using Trizol™ (Life Technologies, Carlsbad, CA, U.S.A.) and cDNA synthesis was carried out using the ImProm-II™ Reverse Transcription System (Promega Corporation, Madison, WI, U.S.A.). Q-PCR was performed using the GoTaq^®^ qPCR Master Mix (Promega Corporation, Madison, WI, U.S.A.) as previously reported [31, 32]. Primer sequences are reported in Supplementary Table S3.

PCR products were detected in the CFX96 Touch Real-Time PCR Detection System (Bio-Rad, Hercules, CA, U.S.A.). Results were analysed using the CFX Maestro Software. A melting curve analysis was performed to discriminate between specific and non-specific PCR products. The relative expression of *GLI1* and *GLI2* mRNA was calculated by using a comparative threshold cycle method and the formula 2^−(DDCt)^ [33]. The level of expression of mRNA of interest was normalized to that of both GAPDH mRNA and 18S rRNA or GAPDH and TBPmRNA.

### Immunofluorescence

HeLa cells were plated on glass coverslips in six-well dishes (1.25 × 10^5^ cells/well) and transfected after 24 h with a total amount of 2 µg of plasmid DNA using Lipofectamine 2000 (Invitrogen, Thermo Fisher Scientific), following manufacturer’s instructions. 24 h after transfection, cells were fixed with 4% paraformaldehyde (10 min, room temperature), permeabilized (0.2% Triton X-100) and incubated with 10% horse serum in PBS/1% BSA for 45 min. Incubation with primary antibody (overnight, 4 °C) and with Cy2- or Cy3-labeled secondary antibodies (40 min, room temperature) was performed. Cell nuclei were labelled with DAPI (Invitrogen, Waltham, MA, USA). Images were taken with a Leica TCS SP8 scanning confocal microscopy system with a Plan Apo 60X objective (Leica Microsystems, Mannheim, Germany).

### Three-Dimensional (3D) spheroid cultures

A375 and SSM2c cells were seeded in DMEM/10% FBS in 96-well plate (1000 cells/well) precoated with 1.5% agarose in water. After 72 h, photos of time 0 were taken and spheroids were treated with drugs or vehicle (DMSO) in DMEM/2.5% FBS. Images were taken after 3 and 6 days of treatment and the volume of the A375 and SSM2c spheroids have been quantified with ImageJ [Volume = 0.5*L*W^2^, L = length (major axis) W = width (minor axis)].

### Statistical analysis

Data are mean ± SD of values obtained from at least three independent experiments. P values were calculated using Student’s t-test (two groups) or one-way analysis of variance (more than two groups; multiple comparison using Bonferroni’s correction. Normal distribution of data has been evaluated. *, *P* ≤ 0.05; **, *P* ≤ 0.01; ***, *P* ≤ 0.001; ****, *P* ≤ 0.0001. Bliss independence analysis was used to evaluate the effects of drug combinations. Bliss score: =0, no functional interaction; >0, synergism; < 0, antagonism.

## Results

### The MEK5/ERK5 pathway positively regulates the transcriptional activity and protein levels of GLI1 and GLI2 in NIH/3T3 cells

**Fig. 1:**
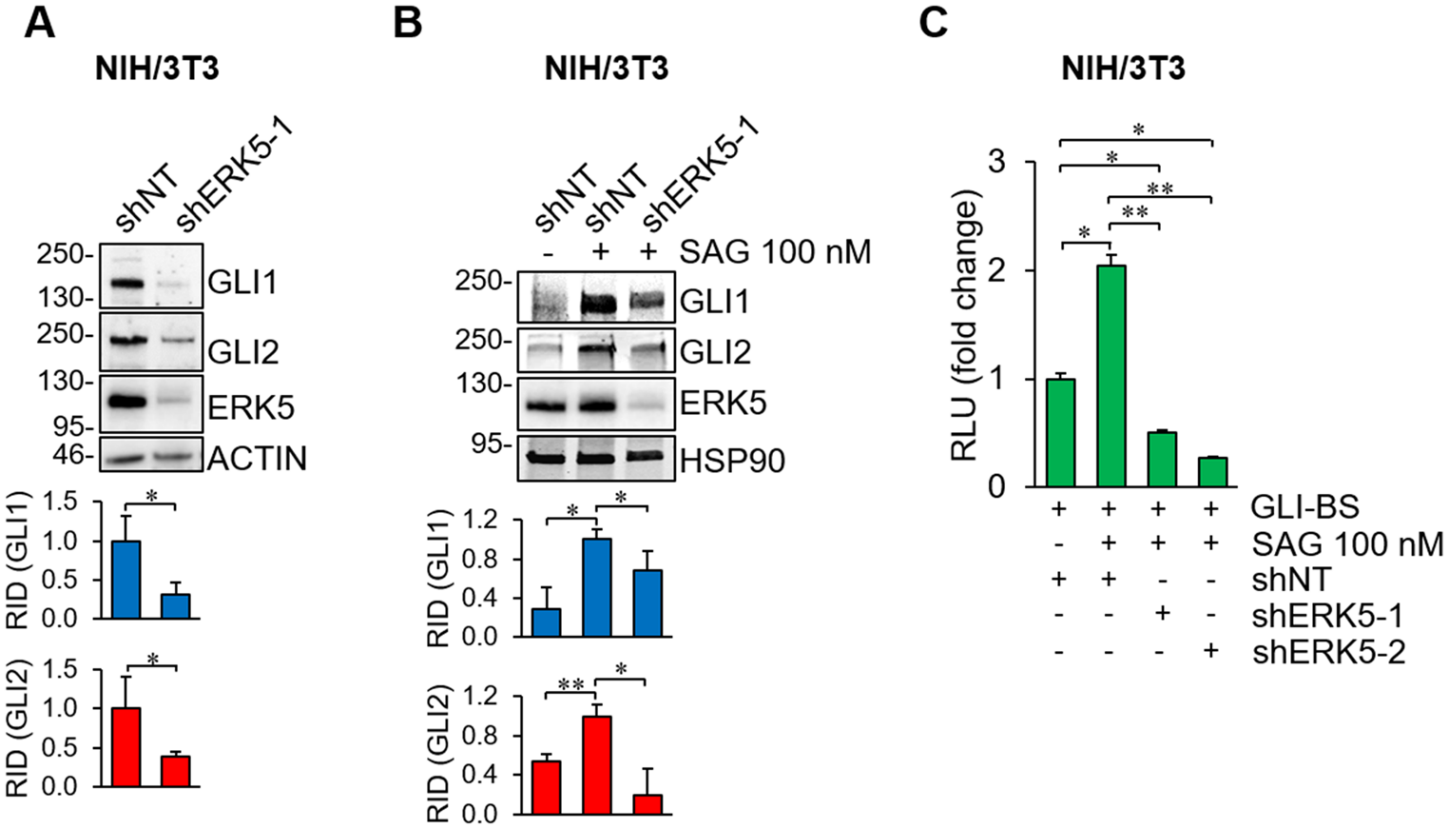
ERK5 silencing inhibits endogenous HH/GLI pathway in NIH/3T3 cells. (**A**-**B**) Cells were transduced with lentiviral vectors carrying control non-targeting shRNA (shNT) or murine ERK5-specific shRNA (shERK5-1), and then serum-deprived for 24 h (**A**) or treated with vehicle (-) or SAG for 48 h (**B**) before lysis and Western Blot. Graphs show averages of relative integrated density (RID) ± SD (*n* = 3). (**C**) Cells transduced as above (shNT, shERK5-1, shERK5-2) were serum-deprived for 24 h, transfected with a GLI responsive luciferase reporter (GLI-BS) for 12 h and treated with vehicle (-) or SAG for 48 h. Relative luciferase activity (RLU) was firefly/Renilla ratios normalized for control. Data are presented as means ± SD (*n* = 3)

**Fig. 2:**
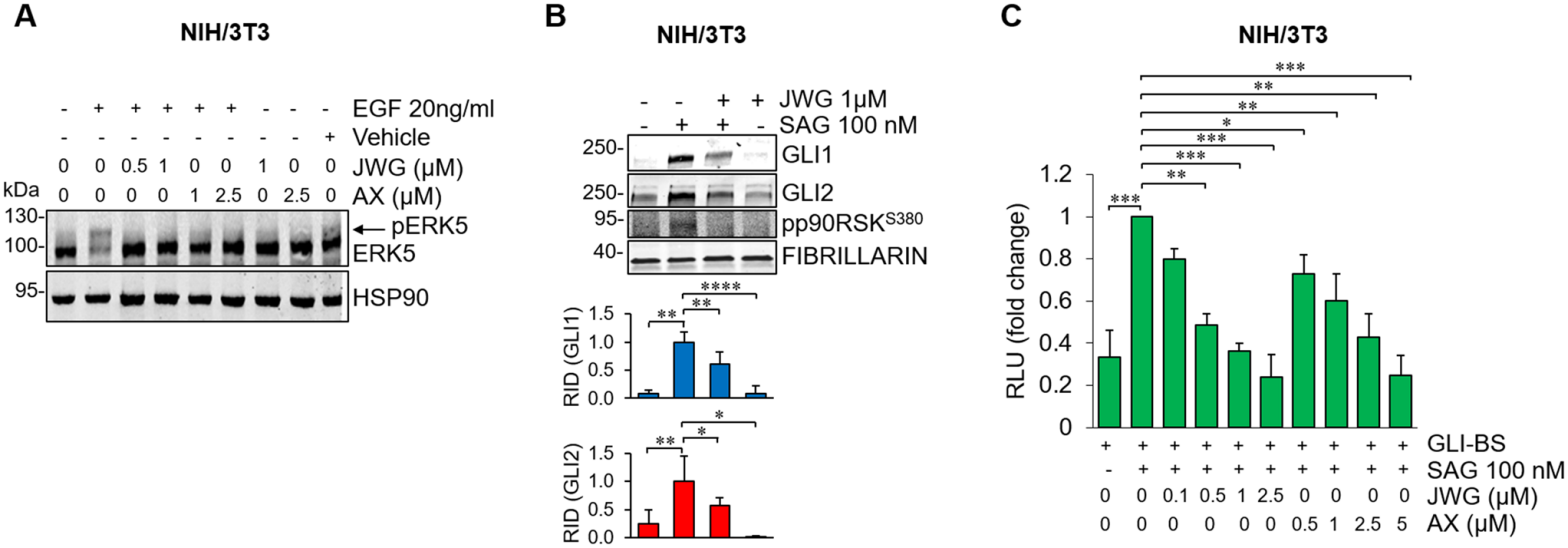
ERK5 inhibition reduces the activity of endogenous HH/GLI pathway in NIH/3T3 cells. (**A**) 24-hour serum-deprived cells were treated with DMSO (Vehicle), JWG-071 (JWG) or AX15836 (AX) at the indicated concentrations for 1 h before exposure to EGF for 10 min. Cell lysates were analysed by Western Blot. The arrow indicates the slower migrating band of phosphorylated ERK5. (**B**) 24-hour serum-deprived cells were treated with SAG and/or JWG-071 (JWG) for 48 h. Cell lysates were analysed by Western Blot. Graphs show averages of relative integrated density (RID) ± SD (*n* = 3). (**C**) 24-hour serum-deprived cells were transfected with a GLI responsive luciferase reporter (GLI-BS) for 12 h and then subjected for 48 h to the indicated treatments. Relative luciferase activity (RLU) was firefly/Renilla ratios normalized for control ± SD (*n* = 3)

To investigate whether the MEK5/ERK5 pathway regulates the HH/GLI signaling, we performed ERK5 silencing using lentiviral vectors expressing murine ERK5-targeting shRNA (shERK5-1/shERK5-2) in NIH/3T3 cells, an established cellular model for the study of HH/GLI signaling (Fig. 1). ERK5 silencing reduced endogenous GLI1 and GLI2 protein levels both in the absence (Fig. 1A) or the presence of the SMO agonist SAG (Fig. 1B). Next, to investigate whether ERK5 affects GLI transcriptional activity, we performed luciferase assays in NIH/3T3 cells using the GLI-binding site luciferase reporter. ERK5 silencing reduced the transcriptional activity of the endogenous HH/GLI pathway induced by SAG (Fig. 1C). Similar results were obtained upon pharmacological inhibition of ERK5 kinase activity using AX15836 and JWG-071 (Fig. 2). These compounds, used at concentrations able to prevent the kinase activity of ERK5 (i.e. autophosphorylation resulting in the slower migration of EGF-induced phosphorylated ERK5, Fig. 2A) reduced GLI1 and GLI2 protein levels (Fig. 2B), and diminished transcriptional activity of the endogenous HH/GLI pathway in a dose-dependent manner compared to cells treated with SAG (Fig. 2C). In keeping with previous observation from our group [14], SAG treatment induced ERK5 activation as confirmed by the increased p90RSK phosphorylation that is an ERK5-regulated downstream target [34], whose expression is decreased upon JWG-071 treatment. The ERK5 inhibitor XMD8-92, used at concentrations that inhibit ERK5 kinase activity with negligible off-target effects [12], was similarly effective in reducing the GLI transcriptional activity induced by SAG (Fig. S1).

**Fig. 3:**
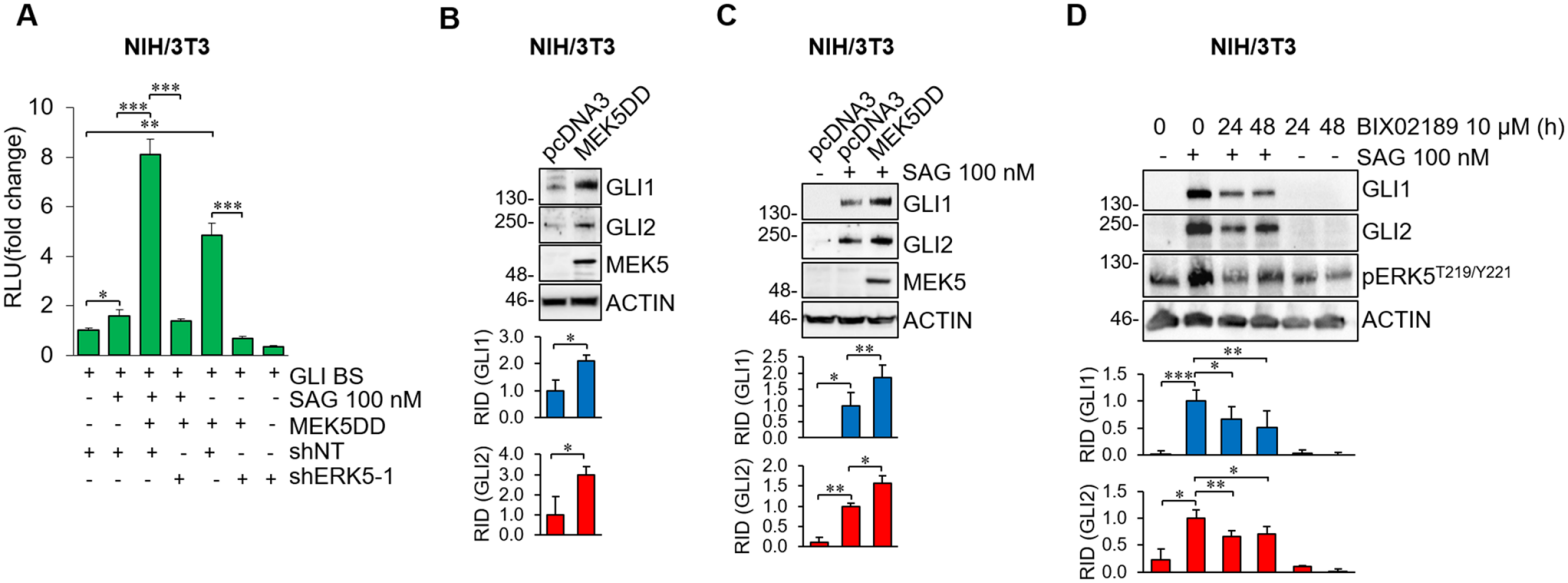
ERK5 activation increases GLI1/GLI2 protein levels and transcriptional activity in NIH/3T3 cells. (**A**) Cells transduced with lentiviral vectors carrying control non-targeting shRNA (shNT) or murine ERK5-specific shRNA (shERK5-1) were transfected with pcDNA3.1 (-) or pCMV5-MEK5DD-HA and with GLI reporter (GLI-BS) for 12 h, before being treated with vehicle (-) or SAG for 48 h. Relative luciferase activity (RLU) was firefly/Renilla ratios normalized for control ± SD (*n* = 3). (**B**-**C**) Cells transfected with pcDNA3.1 or pCMV5-MEK5DD-HA were treated with vehicle (-) or SAG for 48 h. Western Blot was performed. Graphs show averages of relative integrated density (RID) ± SD (*n* = 3). (**D**) 24-hour serum-deprived cells were treated with SAG and/or BIX02189. Western Blot was performed. Graphs show averages of RID ± SD (*n* = 3)

To further confirm that the MEK5/ERK5 pathway positively regulates the HH/GLI signaling, we used a constitutively active form of MEK5 (MEK5DD) to induce activation of endogenous ERK5 in NIH/3T3 cells. MEK5DD expression further increased the transcriptional activity of the endogenous HH/GLI pathway induced by SAG, while ERK5 silencing reverted this effect (Fig. 3A). Likewise, in the absence of SAG, activation of ERK5 by MEK5DD resulted in the marked increase of the basal transcriptional activity of the endogenous HH/GLI pathway, and this effect is hampered by ERK5 silencing (Fig. 3A). Consistently, MEK5DD overexpression increased GLI1 and GLI2 protein levels both in the absence (Fig. 3B) and in the presence of SAG (Fig. 3C), while the MEK5 inhibitor BIX02189 induced a reduction of GLI1 and GLI2 protein levels (Fig. 3D). These results indicate that the MEK5/ERK5 pathway supports HH/GLI signaling activation in NIH/3T3 cells.

### The MEK5/ERK5 pathway positively modulates GLI activity in melanoma cells

**Fig. 4:**
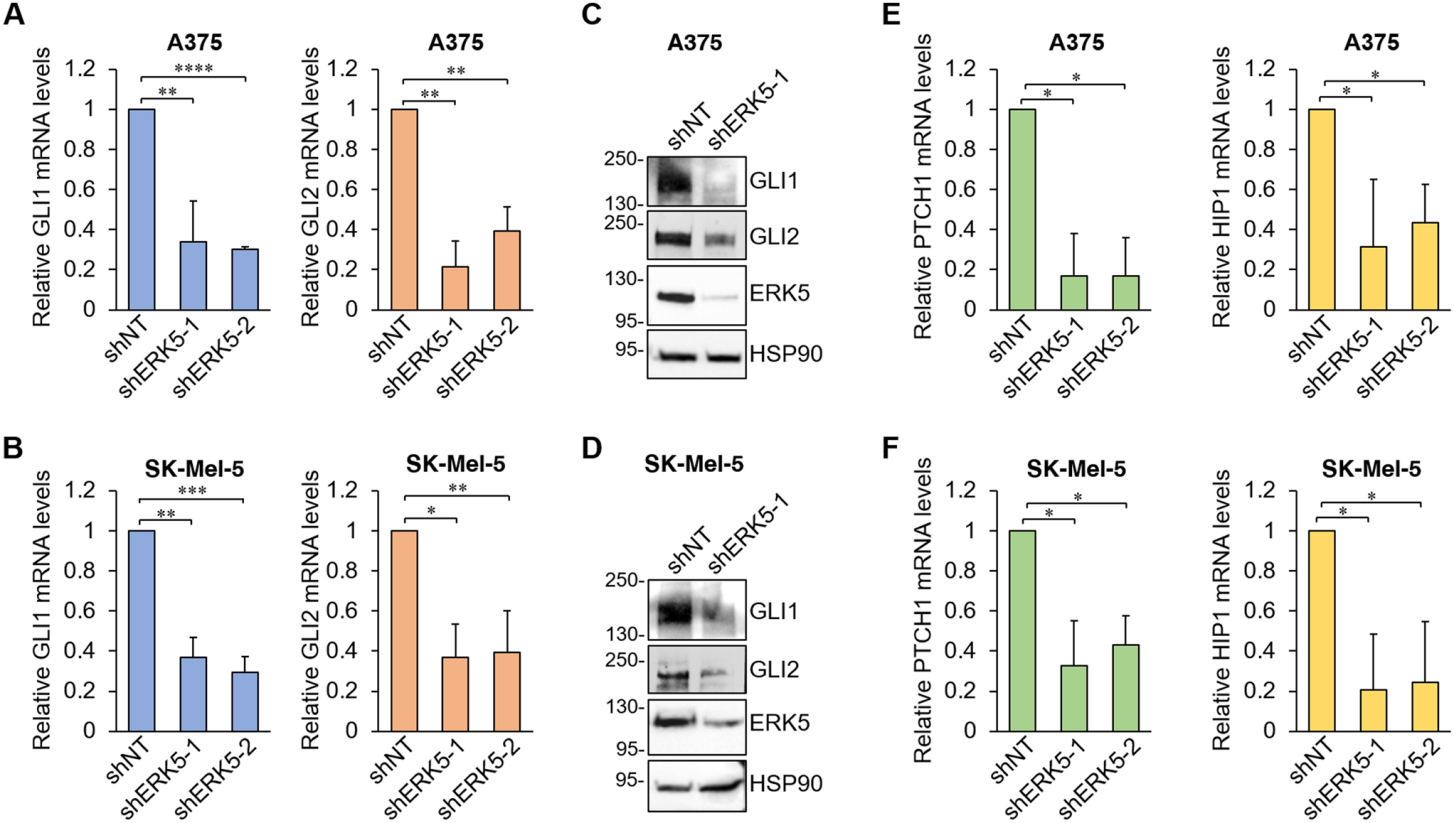
ERK5 silencing reduces mRNA and protein expression levels of GLI1 and GLI2 transcription factors in melanoma cells. (**A**-**B**) A375 (**A**) and SK-Mel-5 (**B**) cells were transduced with lentiviral vectors carrying control non-targeting shRNA (shNT) or human ERK5-specific shRNA (shERK5-1 or shERK5-2). Five days after infection, cells were lysed and GLI1 and GLI2 mRNA levels determined by Q-PCR. Data are presented as means ± SD (*n* = 3). (**C**-**D**) A375 (**C**) and SK-Mel-5 (**D**) transduced with lentiviral vectors carrying shNT or sh-hERK5-1 were lysed and Western Blot was performed. (**E-F**) Cells transduced as above were lysed five days after infection and PTCH1 and HIP1 mRNA levels were determined by Q-PCR. Data are presented as means ± SD (*n* = 3)

We then investigated whether the positive regulation of the HH/GLI pathway by ERK5 occurs in melanoma cells. To assess whether ERK5 modulates the expression of GLI proteins in melanoma cells, ERK5 was silenced using lentiviral vectors expressing shRNAs targeting human ERK5 (shERK5-1/shERK5-2) in A375 and SK-Mel-5 cells (Fig. 4 and Fig. S2A). ERK5 silencing reduced the levels of GLI1 and GLI2 mRNA compared with cells transduced with control lentiviral vector (shNT) in both cell lines (Fig. 4A-B). Consistently, ERK5 silencing drastically decreased GLI1 and GLI2 protein levels as determined by Western Blot analyses (Fig. 4C-D). Q-PCR analysis showed that the expression of two GLI target genes, *PTCH1* and *HIP1*, is significantly reduced by ERK5 silencing (Fig. 4E-F), suggesting that ERK5 regulates not only the expression but also GLI transcriptional activity in melanoma cells. Moreover, silencing either *GLI1* or *GLI2* resulted in downregulation of both GLI1 and GLI2 mRNA levels (Fig. S2B), supporting the notion that GLI1 and GLI2 regulate each other as previously reported [35, 36].

**Fig. 5:**
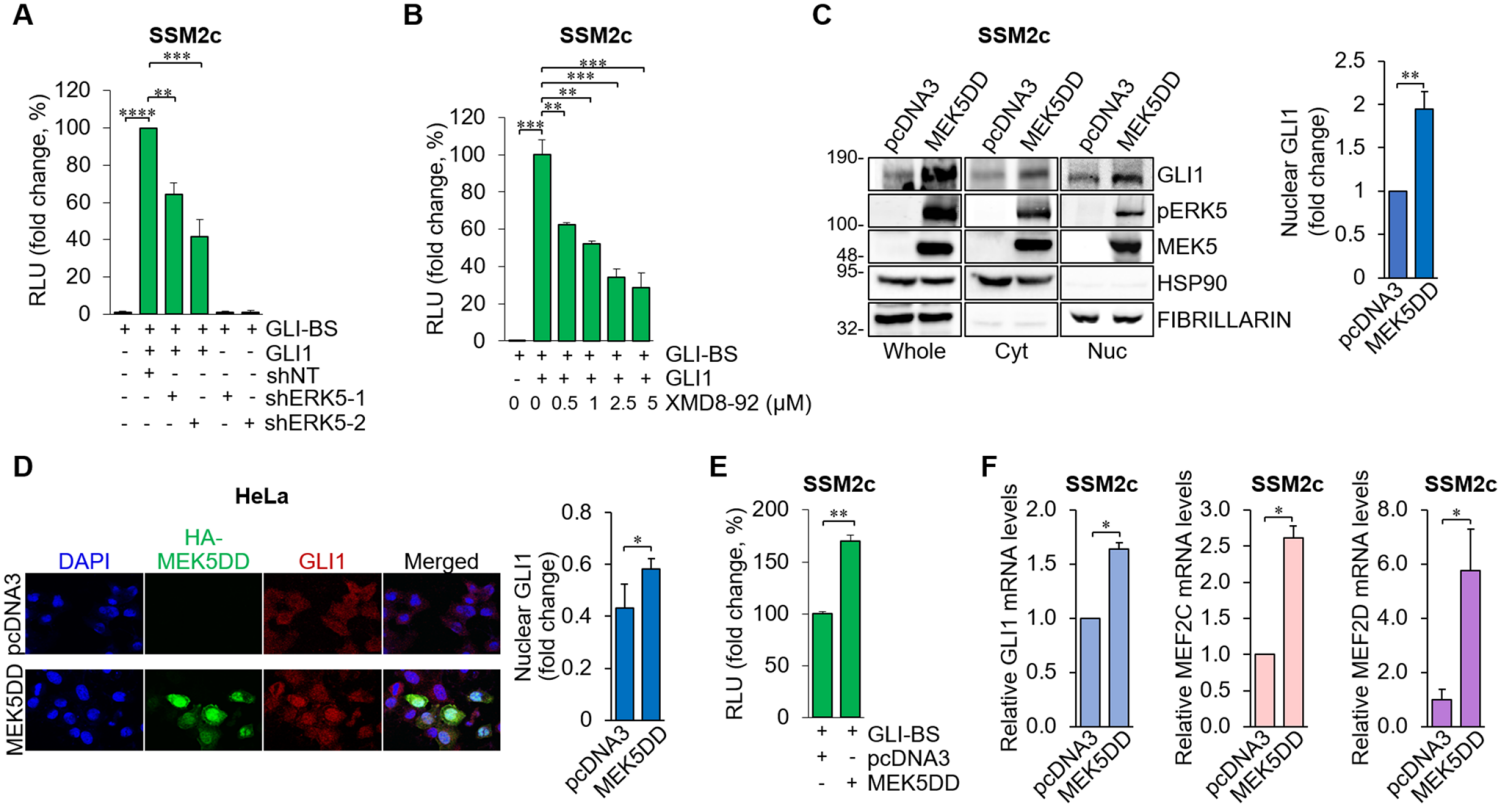
MEK5/ERK5 positively regulates the transcriptional activity of endogenous HH/GLI in melanoma cells. (**A**) Cells transduced with lentiviral vectors carrying control non-targeting shRNA (shNT) or human ERK5-specific shRNA (shERK5-1/shERK5-2) were transfected with pCS2 + MT (-) or pCS2 + MT-GLI1 and GLI-BS for 12 hours. Relative luciferase activity (RLU) was firefly/Renilla ratios normalized for control ± SD (n = 3). (**B**) Cells transfected as above were treated with vehicle (0) or XMD8-92 for 24 hours. RLU was firefly/Renilla ratios normalized for control ± SD (n = 3). (**C**) Cells were transfected with pcDNA3.1 (control) or pCMV5-MEK5DD-HA, lysed after 48 hours, and Western Blot was performed on total (Whole), cytoplasmic (Cyt) or nuclear (Nuc) extracts with the indicated antibodies. Graph shows quantification of nuclear GLI1. (**D**) HeLa cells were transfected with pcDNA3.1 (control) or pCMV5-MEK5DD-HA for 24 hours. Immunofluorescence analysis was performed by staining for HA (HA-MEK5DD, green) and GLI1 (red). Confocal images were analysed to quantify GLI1 nuclear staining, represented in the graph as nuclear Pearson’ Coefficient ± SD (*n* = 3). (**E**) Cells were transfected with pcDNA3.1 (control) or pCMV5-MEK5DD-HA and with a GLI-BS for 12 h. RLU was firefly/Renilla ratios normalized for control ± SD (*n* = 3). (**F**) Cells transfected as above were lysed after 48 h and GLI1, MEF2C and MEF2D mRNA levels were determined by Q-PCR. Data are presented as means ± SD (*n* = 3)

To perform functional studies, we used SSM2c melanoma cells, which have an active HH/GLI pathway [14, 15, 30], and are endowed with higher transfection efficiency compared to A375 and SK-Mel-5 cells. To investigate whether ERK5 modulates GLI1 transcriptional activity, we performed luciferase assays using the GLI-binding site luciferase reporter in SSM2c cells (Fig. 5). Genetic (Fig. 5A) and pharmacologic (Fig. 5B) inhibition of ERK5 reduced the transcriptional activity of ectopic GLI1 in melanoma cells. Furthermore, sustained activation of ERK5 by MEK5DD increased GLI1 protein level and nuclear amount (Fig. 5C). To further evaluate GLI1 intracellular localization upon ERK5 activation by MEK5DD, we used HeLa cells. These cells have been extensively used to study ERK5 signalling [3, 18, 37, 38], and are more suitable for immunofluorescence analysis due to a higher adhesion capacity and lower nucleus/cytoplasm ratio compared to SSM2c cells. Confocal imaging showed that activation of ERK5 by MEK5DD increases GLI1 nuclear localization (Fig. 5D), as well as ERK5 nuclear translocation, as expected (Fig. S3). Moreover, we observed that ERK5 activation by MEK5DD positively modulated endogenous GLI transcriptional activity in SSM2c cells (Fig. 5E). In support of this increase, the analysis of GLI target genes over time (24 and 48 h) showed that MEK5DD induced the expression of PTCH1 and HIP1 mRNA already at 24 h in melanoma cells (Fig. S4A).

Among the transcription factors regulated by MEK5/ERK5, MEF2C is known to control the expression of GLI2 [39]. Here, we observed that ERK5 activation by MEK5DD increased GLI1 and MEF2C mRNA levels, leaving open the possibility that MEF2C may contribute to the regulation of GLI1 expression (Fig. 5F). In the same experimental settings, the mRNA of another well-known target of ERK5, MEF2D, resulted to be increased, as expected (Fig. 5F) [40, 41]. To further investigate the mechanism by which MEK5-ERK5 pathway activates the HH/GLI signaling, SSM2c cells overexpressing MEK5DD were treated with the G/C-specific DNA-binding drug mithramycin A (MTR) to inhibit transcription. Q-PCR analysis showed that MTR treatment did not completely abrogate the increase in PTCH1 expression level induced by MEK5DD (fold change pcDNA3 vs. MEK5DD 1.64; fold change pcDNA3 + MTR vs. MEK5DD + MTR 1.49) (Fig. S4B), suggesting a partial transcriptional regulation. In addition, the expression of endogenous GLI1 protein was further increased by MEK5DD in presence of the proteasome inhibitor MG132 (Fig. S4C). All together, these results indicate that the MEK5/ERK5 pathway positively modulates GLI1 and GLI2 transcription factors both at transcriptional and protein levels in melanoma cells.

### The combination of GLI and MEK5 inhibitors provides enhanced inhibition of melanoma cell growth compared to single treatments

**Fig. 6:**
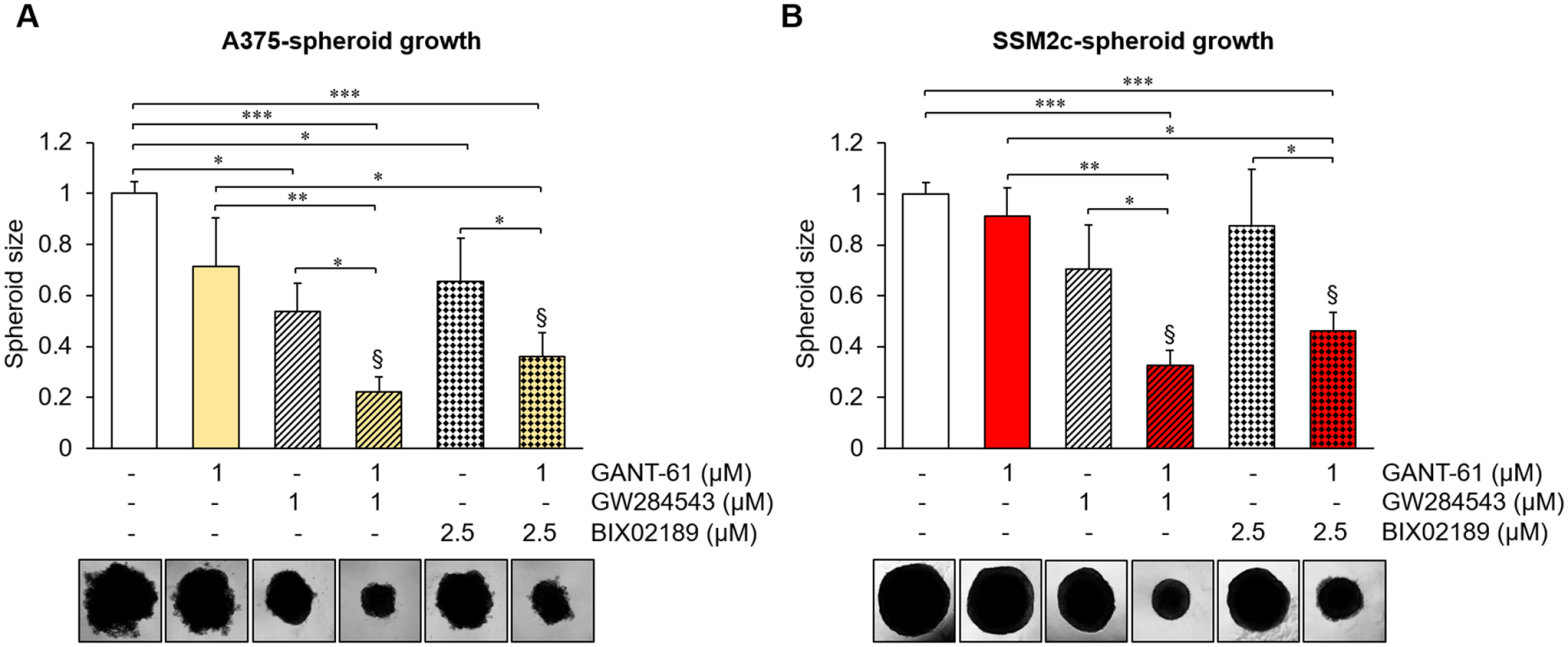
Combined targeting of MEK5 and GLI synergistically reduces the volume of melanoma spheroids. (**A**-**B**) A375 (**A**) and SSM2c (**B**) spheroids were treated with DMSO (-), GANT-61, GW284543 or BIX02189 or with the combinations at the indicated concentrations. Graphs show the quantification of spheroid volume after 5 days normalized for the time point 0. Data represent mean ± SD from three independent experiments. Representative images of spheroids taken at day 5 are shown. § Bliss independence score (> 0) indicates synergistic effects over single treatments. Bliss score = 0.027 (GANT + GW284543) or 0.008 (GANT + BIX02189) in A375 cells; Bliss score = 0.291 (GANT + GW284543) or 0.327 (GANT + BIX02189) in SSM2c cells

We previously showed that co-targeting HH/GLI (GLI1/2 inhibitor GANT-61, SMO inhibitor MRT-92) and MEK5/ERK5 (ERK5 inhibitor XMD8-92 or JWG-071, MEK5 inhibitor BIX02189) pathways elicits significant antitumor activity in melanoma cells, including a drastic reduction of melanoma cell proliferation and colony formation ability [14]. Here, we moved to a 3D model of in vitro tumour growth to test the effects of combined GLI and MEK5 targeting using A375 and SSM2c melanoma spheroids. The combinations of GANT-61 with BIX02189 or with the recently developed MEK5i GW284543 were more effective than single treatments in reducing spheroid growth in both melanoma cell lines used (Fig. 6A-B). Bliss analysis indicated the existence of a synergistic effect in both cell lines.

## Discussion

Aberrant activation of the HH/GLI signaling is associated with several types of cancer, including melanoma [9]. Many studies have also reported non-canonical mechanisms of GLI activation in cancer, which may occur independently of upstream PTCH/SMO signaling [42]. Clinical trials based on the administration of small molecule inhibitors targeting SMO have demonstrated effectiveness in HH-driven tumors, such as advanced basal cell carcinoma [43]. However, the therapeutic efficacy of SMO inhibitors may not be effective in tumors harboring non-canonical activation of GLI transcription factors. We previously showed that ERK5 supports the growth of melanoma cells in vitro and in vivo [12]. Additionally, we have recently reported that ERK5 is required for the HH/GLI-dependent melanoma cell proliferation and that GLI1, the major downstream effector of the HH/GLI signaling, positively regulates ERK5 expression [14]. In this study, we show that the MEK5/ERK5 pathway promotes the activation of HH/GLI signaling in melanoma cells, providing a novel mechanism of activation of GLI transcription factors.

A number of studies reported the existence of functional interactions between the HH/GLI and ERK1/2 pathways, that affect a diverse array of processes relevant for cancer, including cell proliferation, escape from apoptosis, cell migration, local invasiveness as well as metastasis formation [10]. Our results here show that MEK5/ERK5 signaling acts upstream of the GLI transcription factors, thus contributing to their expression and activity. Indeed, we found that both genetic and pharmacologic inhibition of ERK5 inhibits GLI transcriptional activity, and reduces GLI mRNA and protein levels in either untransformed immortalized mouse fibroblasts and melanoma cells. In further support of a positive regulation of the HH/GLI signaling by the MEK5/ERK5 pathway, ERK5 activation by overexpression of a constitutively active MEK5 mutant resulted in an increase of GLI transcriptional activity and nuclear localization in melanoma cells. Despite several lines of evidence of the regulation of GLI1/2 activities by MEK5/ERK5 the underlying mechanisms remain to be elucidated, although our data suggest a regulation at transcriptional and protein levels.

Another important outcome of this study is that the combination of MEK5 inhibitors with GLI inhibitors emerged as a potential therapeutic strategy. Indeed, the combinations GANT-61 + GW284543 or GANT-61 + BIX02189 were more effective than either drug alone in reducing melanoma spheroid volume. These results obtained using a 3D growth model, add value to and further support what was previously published by our group in melanoma cell lines, in which ERK5 (JWG-071 and XMD8-92) or MEK5 (BIX02189) inhibitors synergize with SMO or GLI inhibitors (MRT-92 and GANT61) in reducing colony formation ability and proliferation in 2D cultures [14]. In light of future clinical trials combining GLI and MEK5/ERK5 inhibitors, it will be equally important to identify possible biomarkers of activation of HH/GLI and MEK5/ERK5 pathways to identify the subset of cancers that will likely respond to such inhibitors and to monitor the efficacy of the therapy.

## Conclusions

In summary, we provide evidence that the MEK5/ERK5 pathway promotes activation of the HH/GLI signaling in melanoma cells and that combined targeting of MEK5/ERK5 and HH/GLI signaling pathways might be a promising therapeutic strategy for a subset of melanomas harboring activation of both pathways.

## Electronic supplementary material

Below is the link to the electronic supplementary material.

Supplementary Material 1

Supplementary Material 2

Supplementary Material 3

Supplementary Material 4

Supplementary Material 5

Supplementary Material 6

Supplementary Material 7

Supplementary Material 8

## Data Availability

No datasets were generated or analysed during the current study.

## Abbreviations

BMK1: Big mitogen-activated kinase 1
DMEM: Dulbecco’s modified Eagle’s medium
EGF: Epidermal growth factor
ERK5: Extracellular signal-regulated kinase 5
FBS: Foetal bovine serum
GLI-BS: GLI-binding site
HH: Hedgehog
MAPK: Mitogen-activated protein kinase
PTCH: Patched
RID: Relative integrated density
RLU: Relative luciferase activity
SD: Standard deviation
SMO: Smoothened
