# Supplementary material for: The MEK5/ERK5 pathway promotes the activation of the Hedgehog/GLI signaling in melanoma cells
Source: Cell Oncol (Dordr). 2025 Feb 25;48(3):789–99. doi: 10.1007/s13402-025-01050-z (PMC12119679; doi:10.1007/s13402-025-01050-z)
Supplement: Supplementary file 1 — Supplementary Material 1 [file 13402_2025_1050_MOESM1_ESM.docx]

**The MEK5/ERK5 pathway promotes the activation of the Hedgehog/GLI signaling in melanoma cells**

Ignazia Tusa^1,*^, Sinforosa Gagliardi^2^, Alessio Menconi^1^, Luisa Maresca^1,2^, Alessandro Tubita^1^, Matteo Lulli^1^, Barbara Stecca^2,*, ^^, Elisabetta Rovida^1,*, ^^

^1^Department of Experimental and Clinical Biomedical Sciences "Mario Serio", University of Florence, 50134 Florence, Italy; ignazia.tusa@unifi.it, [alessio.menconi@unifi.it](mailto:alessio.menconi@unifi.it), [alessandro.tubita@unifi.it](mailto:alessandro.tubita@unifi.it), [matteo.lulli@unifi.it](mailto:matteo.lulli@unifi.it), elisabetta.rovida@unifi.it

^2^Core Research Laboratory - Institute for Cancer Research and Prevention (ISPRO), Florence, Italy; sinforosa.gagliardi@unisalento.it, b.stecca@ispro.toscana.it

^*^Corresponding authors: [ignazia.tusa@unifi.it](mailto:ignazia.tusa@unifi.it); [elisabetta.rovida@unifi.it](mailto:elisabetta.rovida@unifi.it); [b.stecca@ispro.toscana.it](mailto:b.stecca@ispro.toscana.it)

^^^Shared senior authorship

**Cell lines and treatments**

The human melanoma cell line MeWo was obtained from ATCC (Manassas, VA, U.S.A.). Cell were maintained in Dulbecco’s modified Eagle’s medium (DMEM) supplemented with 10% heat-inactivated foetal bovine serum (FBS), 2 mM glutamine, 50 U/mL penicillin, and 50 mg/mL streptomycin (Euroclone, Milan, Italy), and incubated at 37 °C in a water-saturated atmosphere containing 95% air (21% O_2_) and 5% CO_2_. MG132 (Sigma-Aldrich, St Louis, MO, U.S.A.) and mithramycin (Merck Millipore, Burlinghton, MA) were dissolved in DMSO.

**RNA interference**

Lentiviruses were produced in HEK-293T cells as previously reported [1]. Lentiviral vectors for stable knockdown of *GLI1* or *GLI2* in human melanoma cells were TRC1.5-pLKO.1-puro vector containing shRNA sequences (shNT), or target-specific sequences. For the sequences of the shRNA used see Supplementary Table S2. Transduced cells were selected with 2 µg/ml puromycin for at least 72 hours.


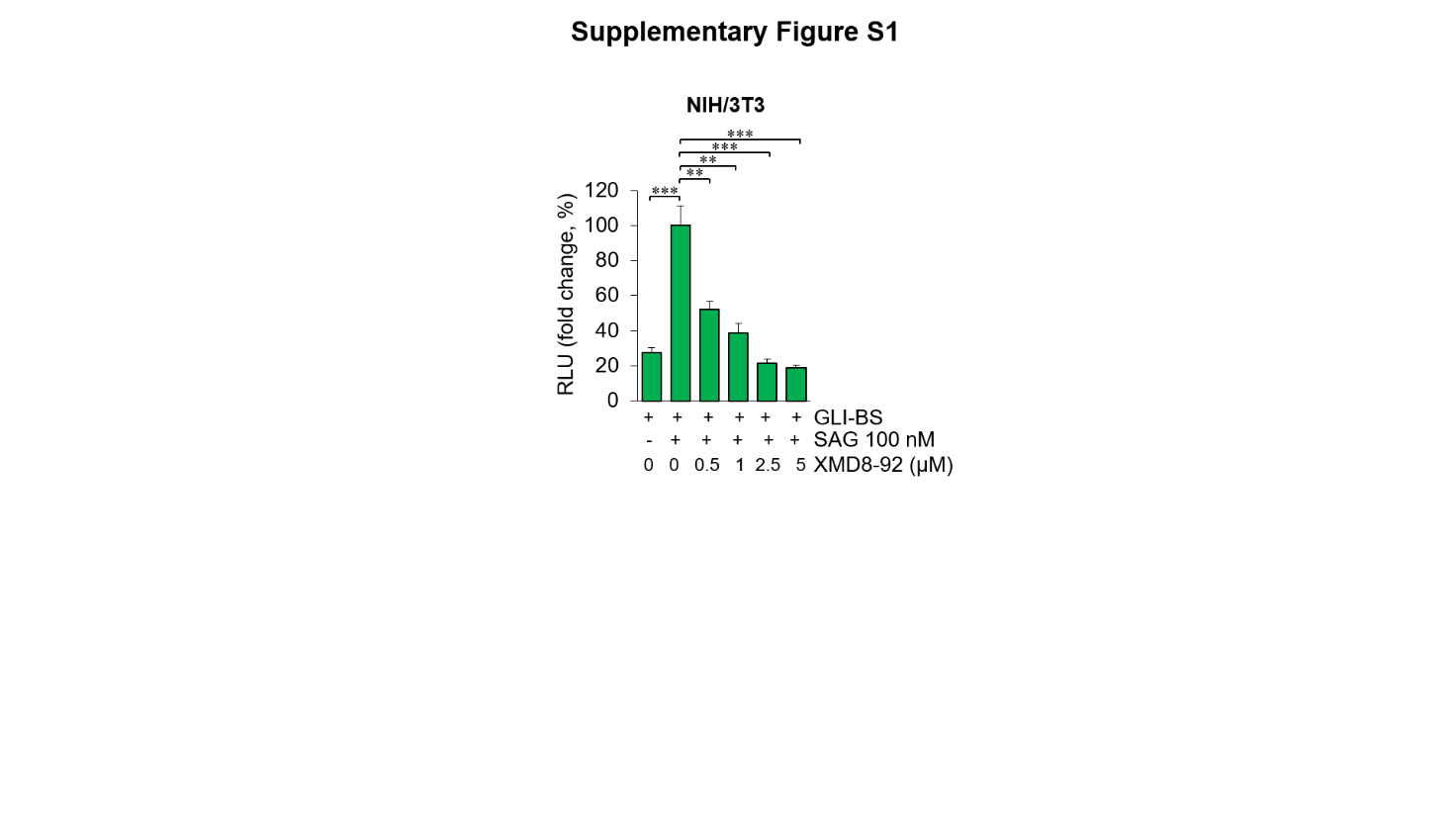
**SUPPLEMENTARY FIGURES**

**Fig S1.** XMD8-92 ERK5 inhibitor reduces the transcriptional activity of endogenous HH/GLI pathway in NIH/3T3 cells. NIH/3T3 cells were serum-deprived for 24 hours, transfected with a GLI responsive luciferase reported (GLI-BS) for 12 hours and then subjected for 48 hours to the indicated treatments. Luciferase assay for GLI transcriptional activity was then performed. Relative luciferase activity (RLU) was firefly/Renilla ratios normalized for control (cells treated with SAG) ± SD (n =3). **, *P* ≤ 0.01; ***, *P* ≤ 0.001.


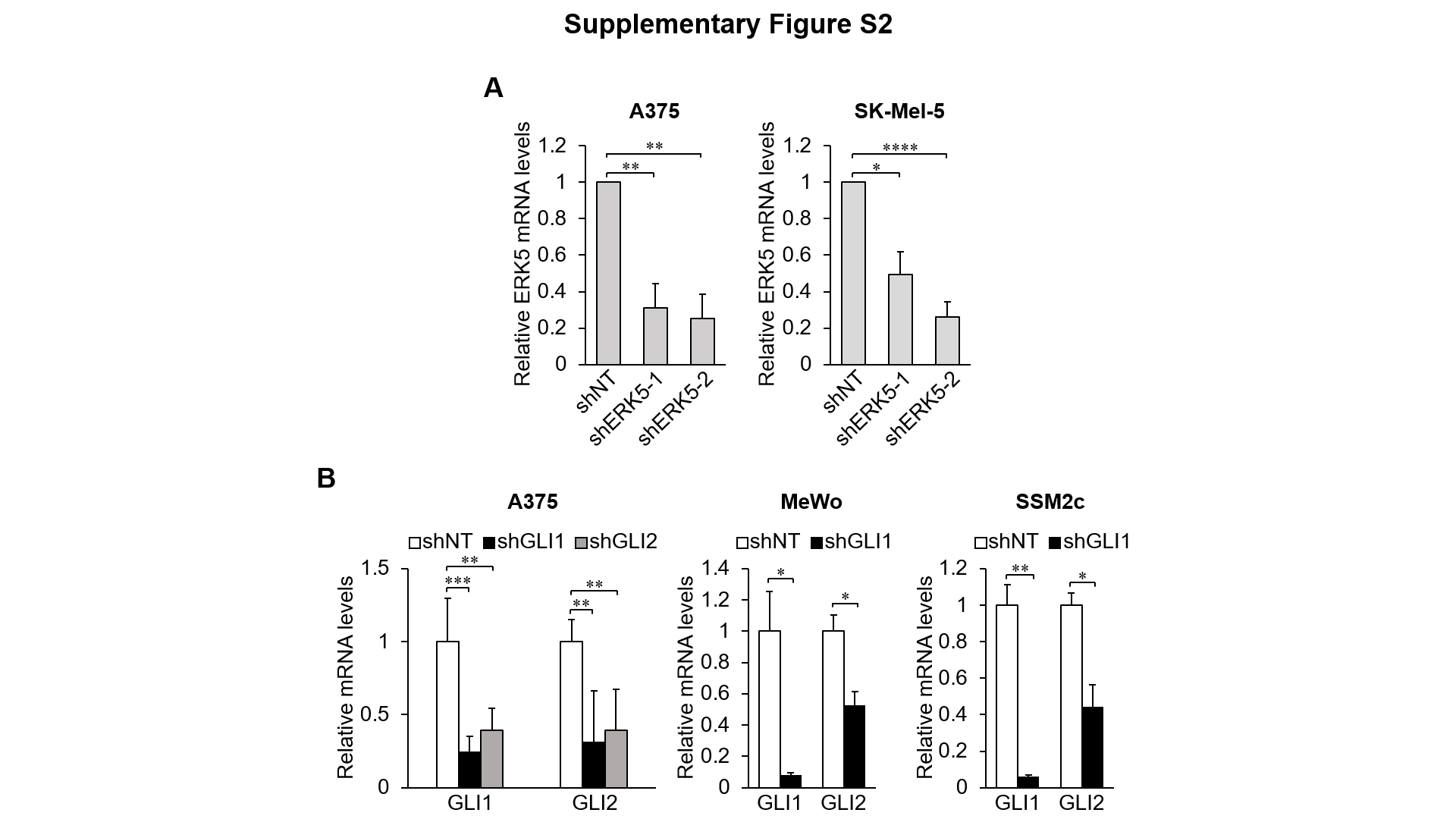


**Fig S2.** ERK5, GLI1 and GLI2 silencing in melanoma cells. (**A**) A375 and SK-Mel-5 cells were transduced with lentiviral vectors carrying control non-targeting shRNA (shNT) or human ERK5-specific shRNA (shERK5-1 or shERK5-2). Five days after infection, cells were lysed and ERK5 mRNA levels determined by Q-PCR. Data are presented as means ± SD (n=3). *, *P* ≤ 0.05; **, *P* ≤ 0.01; ****, *P* ≤ 0.0001. (**B**) A375, MeWo and SSM2c cells were transduced with lentiviral vectors carrying control non-targeting shRNA (shNT) or human GLI1- or GLI2-specific shRNA (shGLI1 or shGLI2). Five days after infection, cells were lysed and GLI1 or GLI2 mRNA levels were determined by Q-PCR. Data are presented as means ± SD (n=3). *, *P* ≤ 0.05; **, *P* ≤ 0.01; ***, *P* ≤ 0.001.


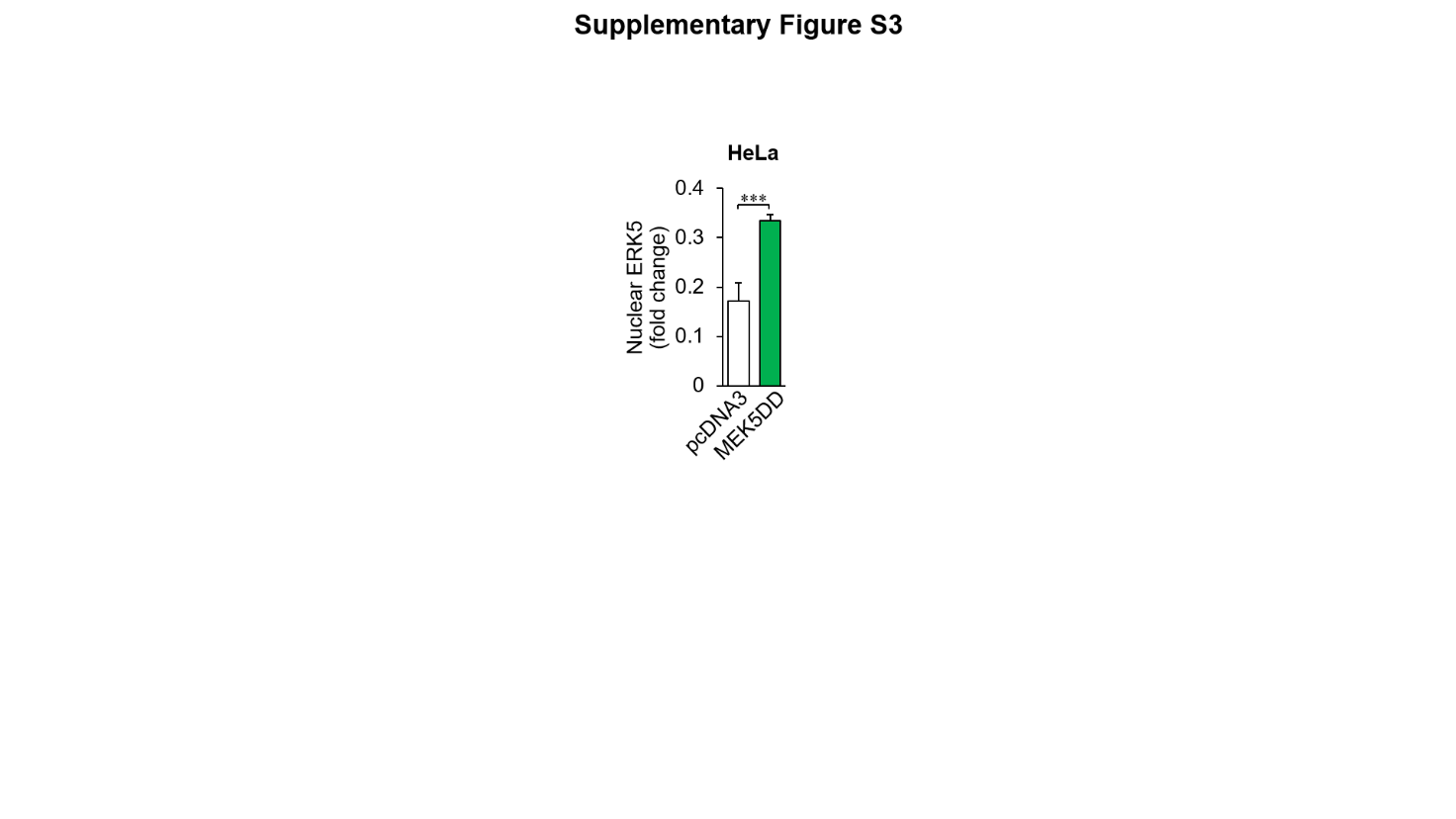


**Fig S3.** Effect of MEK5DD overexpression on ERK5 intracellular localization. HeLa cells were transfected with pcDNA3.1 (as control) or pCMV5-MEK5DD-HA for 24 hours, and immunofluorescence was performed. Confocal images were analysed to quantify ERK5 nuclear staining, represented in the graph as nuclear Pearson’ Coefficient ± SD (n = 3). ***, *P* ≤ 0.001.


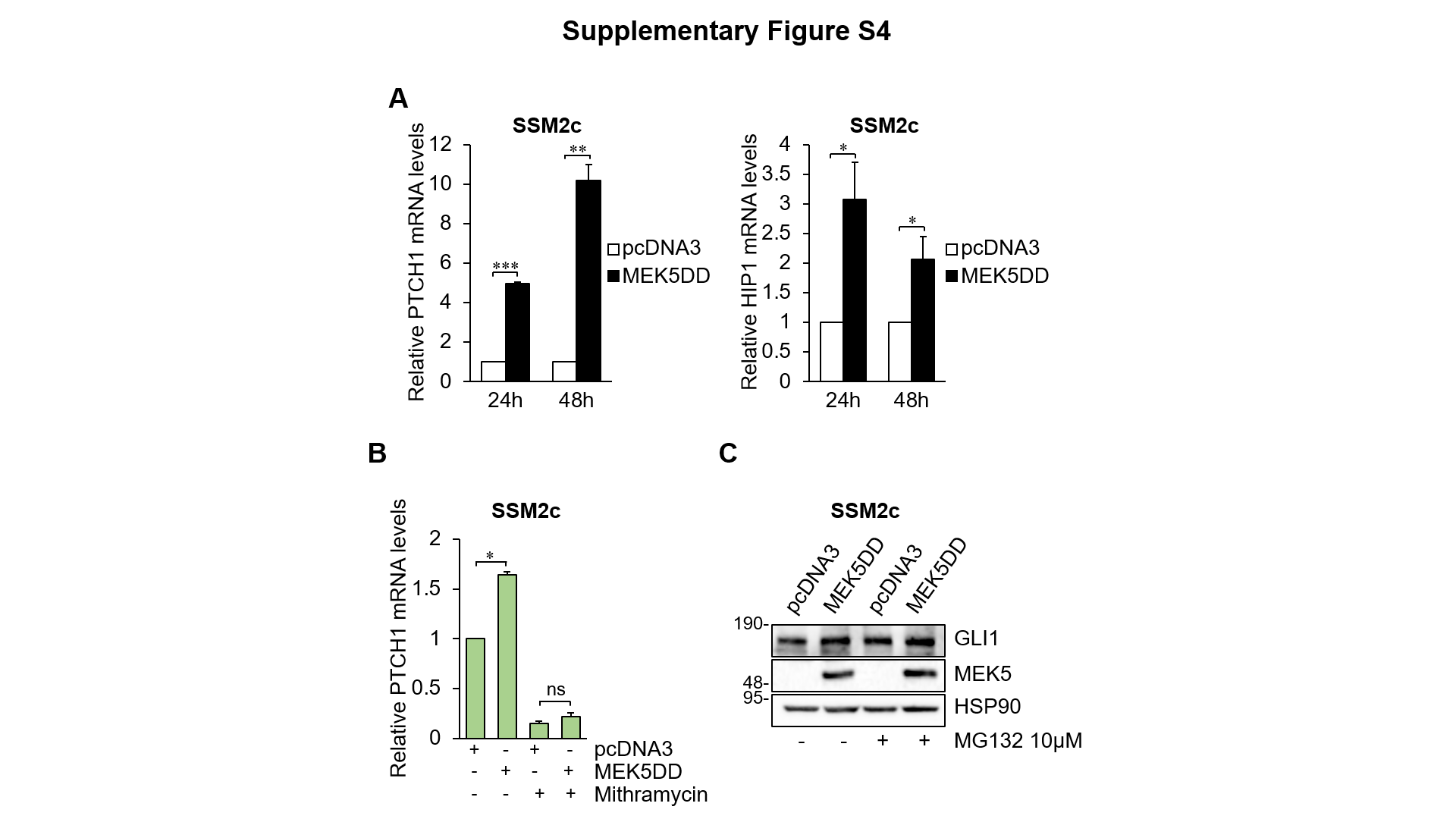
**Fig S4.** Effect of ERK5 activation on the expression of GLI-target genes and mechanism of ERK5-dependent regulation of GLI **(A)** SSM2c cells were transfected with pcDNA3.1 (as control) or pCMV5-MEK5DD-HA. 24 or 48 hours after transfection, cells were lysed and PTCH1 and HIP1 mRNA levels determined by Q-PCR. Data are presented as means ± SD (n=3). *, *P* ≤ 0.05; **, *P* ≤ 0.01; ***, *P* ≤ 0.001. **(B)** Cells transfected as above were treated for 16 hours with mithramycin 200nM, lysed and PTCH1 mRNA levels determined by Q-PCR. Data are presented as means ± SD (n=3). *, *P* ≤ 0.05. **(C)** Cells transfected as above were treated 4 hours with MG132 10 µM, lysed and Western Blot was then performed with the indicated antibodies.
