# Supplementary figures and images for: The MEK5/ERK5 pathway promotes the activation of the Hedgehog/GLI signaling in melanoma cells
Source: Cell Oncol (Dordr). 2025 Feb 25;48(3):789–99. doi: 10.1007/s13402-025-01050-z (PMC12119679; doi:10.1007/s13402-025-01050-z)

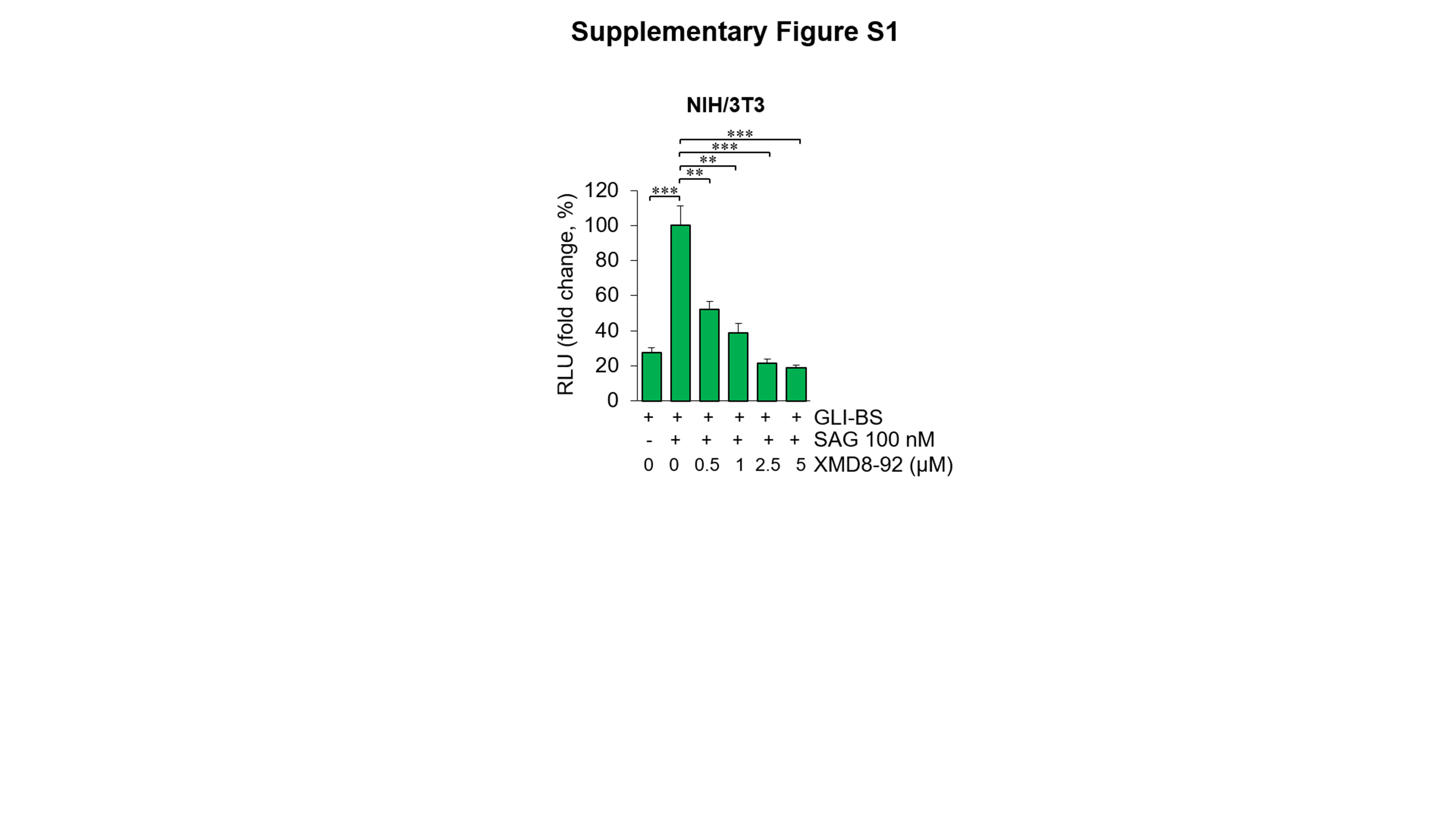

Supplement: Supplementary file 2 — Supplementary Material 2 [file 13402_2025_1050_MOESM2_ESM.tif]

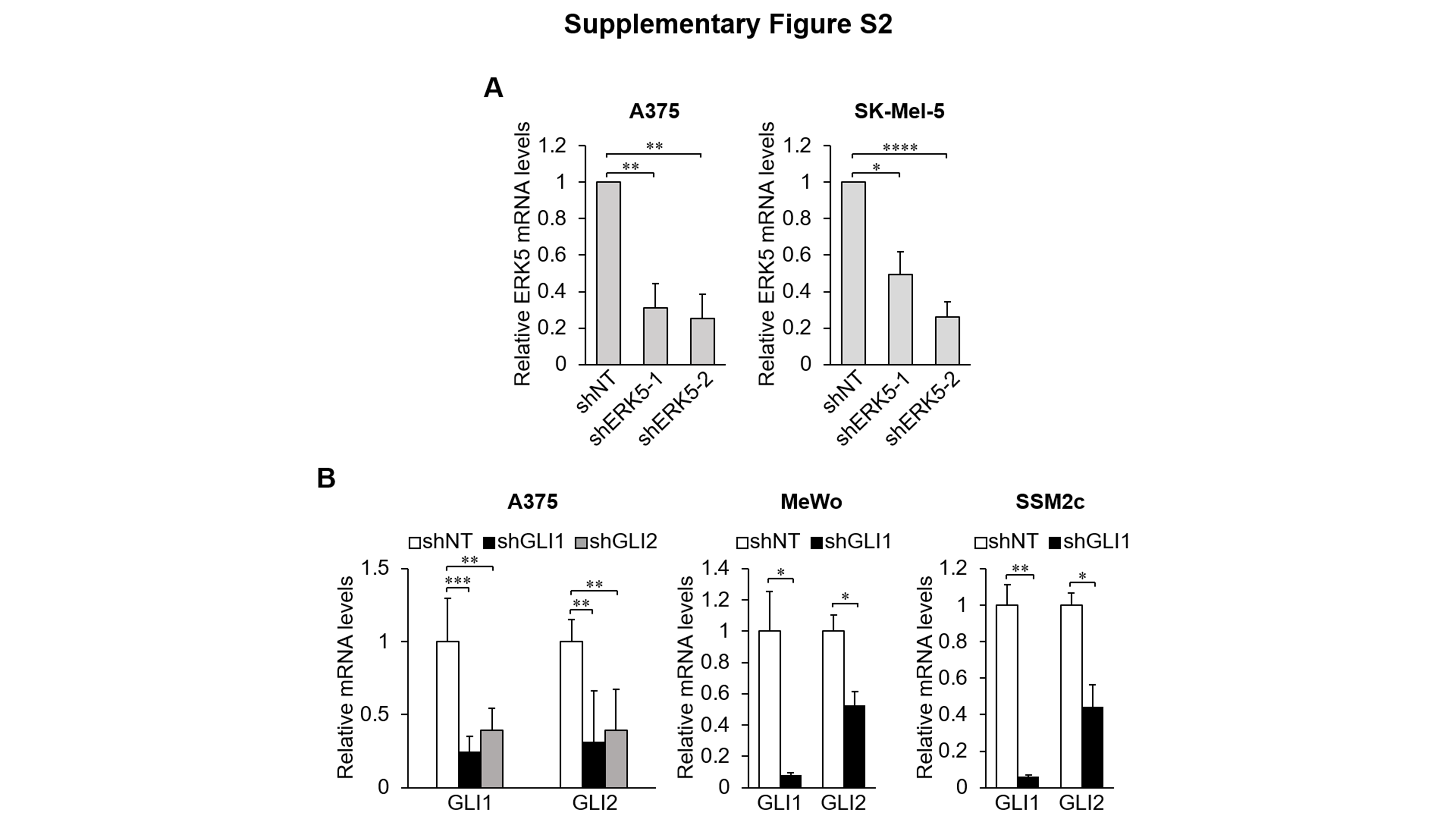

Supplement: Supplementary file 3 — Supplementary Material 3 [file 13402_2025_1050_MOESM3_ESM.tif]

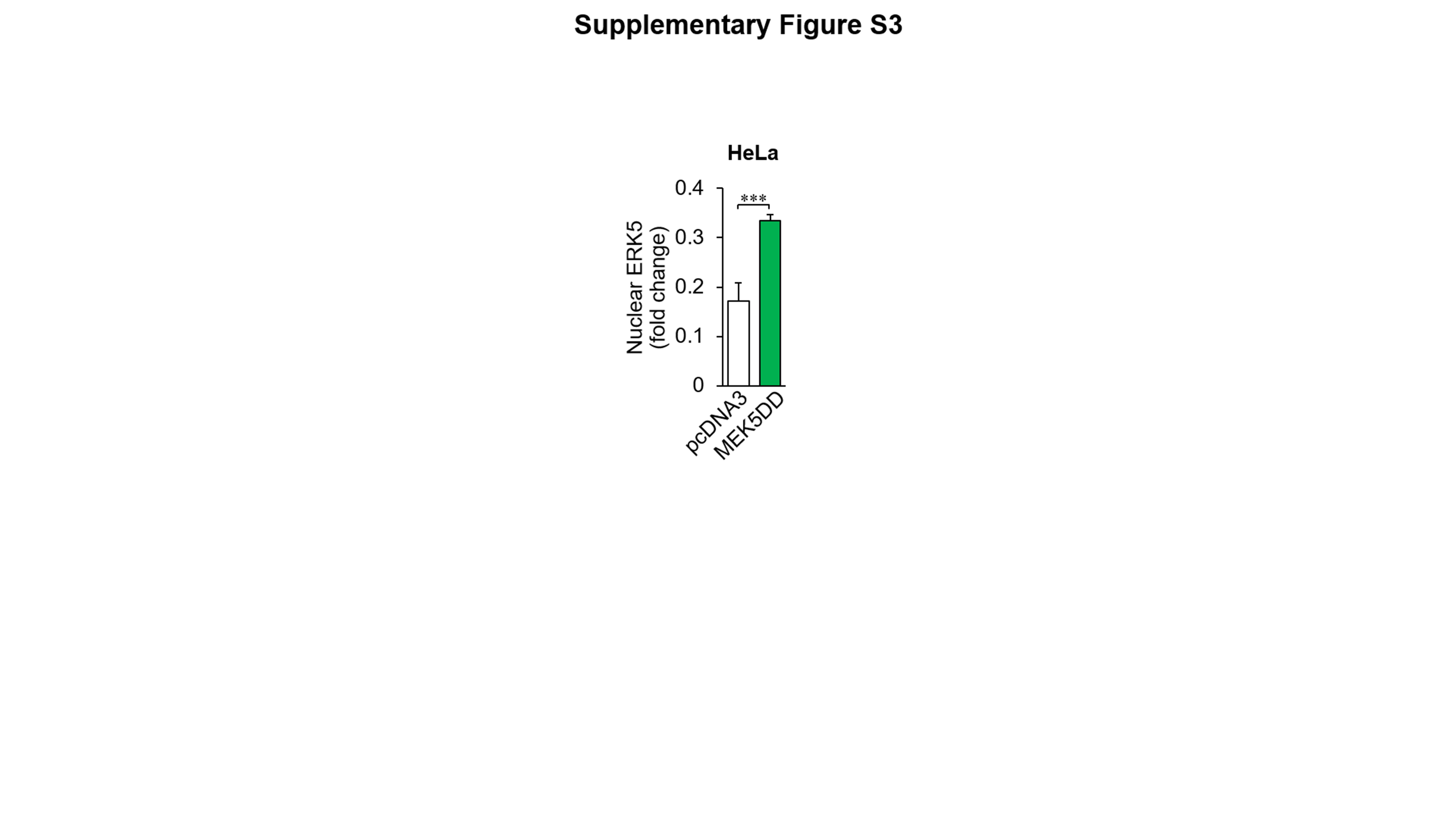

Supplement: Supplementary file 4 — Supplementary Material 4 [file 13402_2025_1050_MOESM4_ESM.tif]

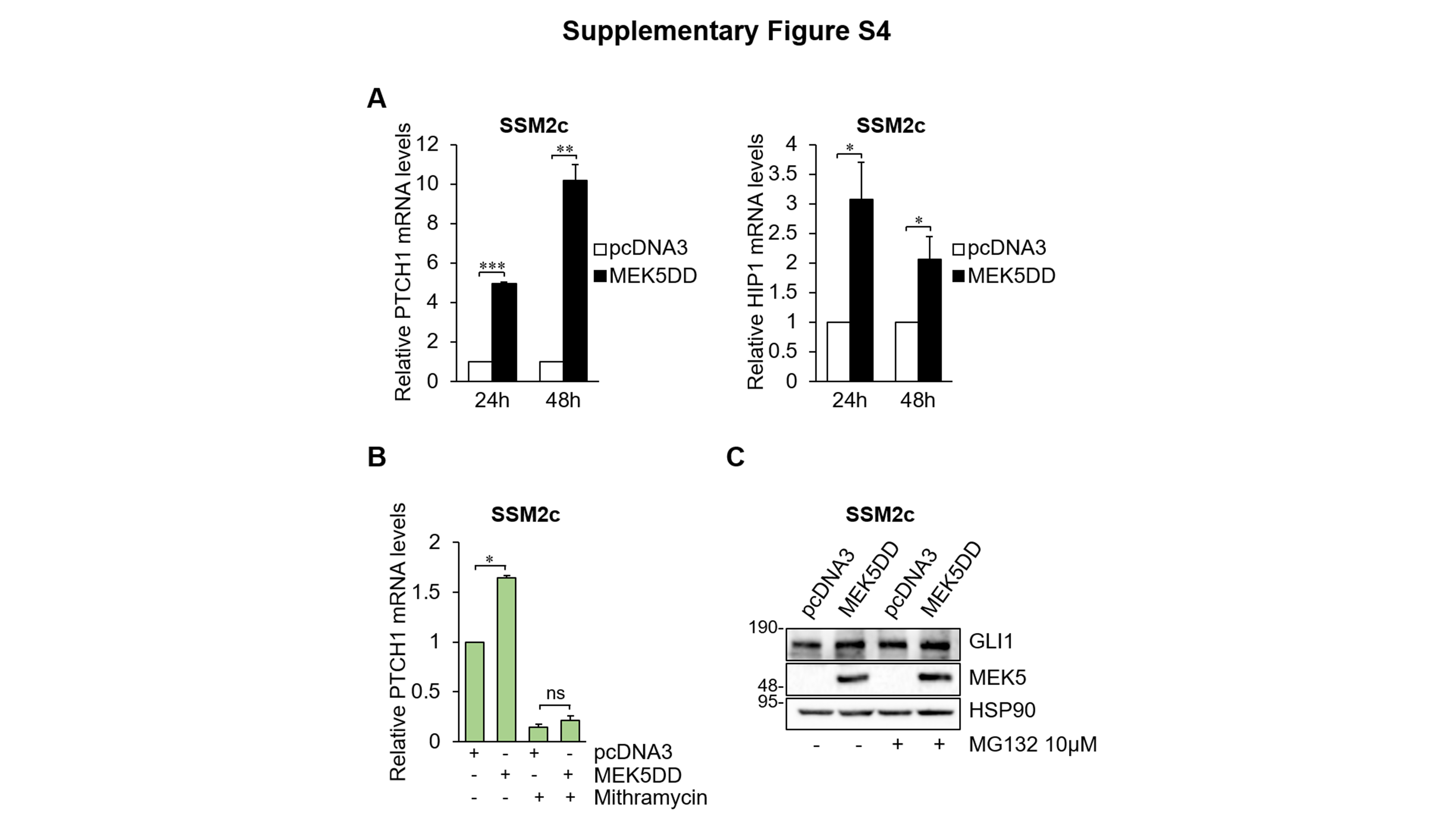

Supplement: Supplementary file 5 — Supplementary Material 5 [file 13402_2025_1050_MOESM5_ESM.tif]
