# Supplementary material for: The MEK5/ERK5 pathway promotes the activation of the Hedgehog/GLI signaling in melanoma cells
Source: Cell Oncol (Dordr). 2025 Feb 25;48(3):789–99. doi: 10.1007/s13402-025-01050-z (PMC12119679; doi:10.1007/s13402-025-01050-z)
Supplement: Supplementary file 6 — Supplementary Material 6 [file 13402_2025_1050_MOESM6_ESM.docx]

| **Protein** | **Use** | **Source** | **Notes** | **Cat. No.** | **Company** |
| --- | --- | --- | --- | --- | --- |
| GLI1 | WB/IF | Mouse monoclonal | L42B10 | #2643 | Cell Signaling Technology, Danvers, MA, USA |
| GLI2 | WB | Mouse polyclonal |  | AF3635 | R&D Systems, Minneapolis, MN, USA |
| ERK5 | WB | Rabbit polyclonal |  | #3372 | Cell Signaling Technology, Danvers, MA, USA |
| ERK5 | IF | Mouse monoclonal | C-7 | sc-398015 | Santa Cruz Biotechnology, Santa Cruz, CA, USA |
| pERK5-T218/Y220 | WB | Rabbit polyclonal |  | #3371 | Cell Signaling Technology, Danvers, MA, USA |
| MEK5 | WB | Goat polyclonal | C-20 | sc-1287 | Santa Cruz Biotechnology, Santa Cruz, CA, USA |
| pp90RSK-S380 | WB | Rabbit polyclonal |  | #9341 | Cell Signaling Technology, Danvers, MA, USA |
| HA | IF | Rabbit  monoclonal |  | #13919 | Cell Signaling Technology, Danvers, MA, USA |
| Actin | WB | Mouse monoclonal | AC-15 | A1978 | Sigma-Aldrich St. Louis, MO, USA |
| HSP90 | WB | Mouse monoclonal |  | sc-13119 | Santa Cruz Biotechnology, Santa Cruz, CA, USA |
| Fibrillarin | WB | Mouse monoclonal |  | sc-374022 | Santa Cruz Biotechnology, Santa Cruz, CA, USA |
| IRDye 800CW | WB | Goat anti-rabbit |  | 926-32211 | LI-COR Biosciences, Lincoln, NE, USA |
| IRDye 800CW | WB | Goat anti-mouse |  | 926-32210 | LI-COR Biosciences, Lincoln, NE, USA |
| IRDye 800CW | WB | Donkey anti-goat |  | 926-32214 | LI-COR Biosciences, Lincoln, NE, USA |
| IRDye 680RD | WB | Goat anti-rabbit |  | 926-68071 | LI-COR Biosciences, Lincoln, NE, USA |
| IRDye 680RD | WB | Goat anti-mouse |  | 926-68070 | LI-COR Biosciences, Lincoln, NE, USA |
| IRDye 680RD | WB | Donkey anti-goat |  | 926-68074 | LI-COR Biosciences, Lincoln, NE, USA |

**Supplementary Table S1. List of the antibodies used and their application.**
