# Supplementary material for: The MEK5/ERK5 pathway promotes the activation of the Hedgehog/GLI signaling in melanoma cells
Source: Cell Oncol (Dordr). 2025 Feb 25;48(3):789–99. doi: 10.1007/s13402-025-01050-z (PMC12119679; doi:10.1007/s13402-025-01050-z)
Supplement: Supplementary file 7 — Supplementary Material 7 [file 13402_2025_1050_MOESM7_ESM.docx]

| **Gene** | **Reference sequence** | **shRNA** | **Clone number** | **Sense sequence 5’ to 3’** |
| --- | --- | --- | --- | --- |
| None mammalian |  | shNT | SHC202 | CCGGCAACAAGATGAAGAGCACCAACTC-GAGTTGGTGCTCTTCATCTTGTTGTTTTT |
| *Erk5* | NM_011841 | shERK5-1 | TRCN0000023236 | CCGGCGACAATATCATCGCCATCAACTCGAGTTGATGGCGATGATATTGTCGTTTTT |
| *Erk5* | NM_011841 | shERK5-2 | TRCN0000232400 | CCGGAGACCCACCTTTCAGCCTTAACTCGAGTTAAGGCTGAAAGGTGGGTCTTTTTTG |
| *ERK5* | NM_139032 | shERK5-1 | TRCN0000010262 | CCGGGCTGCCCTGCTCAAGTCTTTGCTCGAGCAAAGACTTGAGCAGGGCAGCTTTTT |
| *ERK5* | NM_139032 | shERK5-2 | TRCN0000010275 | CCGGGCCAAGTACCATGATCCTGATCTCGAGATCAGGATCATGGTACTTGGCTTTTT |
| *GLI1* | NM_005269 | shGLI1 | TRCN000020485 | CCGGCCTGATTATCTTCCTTCAGAACTCGAGTTCTGAAGGAAGATAATCAGGTTTTT |
| *GLI2* | NM_001374353 | shGLI2 | TRCN0000033329 | CCGGCCGCTTCAGATGACAGATGTTCTCGAGAACATCTGTCATCTGAAGCGGTTTTTG |

**Supplementary Table S2. List and sequences of the shRNAs used in the study.**
