# Supplementary material for: The MEK5/ERK5 pathway promotes the activation of the Hedgehog/GLI signaling in melanoma cells
Source: Cell Oncol (Dordr). 2025 Feb 25;48(3):789–99. doi: 10.1007/s13402-025-01050-z (PMC12119679; doi:10.1007/s13402-025-01050-z)
Supplement: Supplementary file 8 — Supplementary Material 8 [file 13402_2025_1050_MOESM8_ESM.docx]

| **Gene** | **Primer sequence (5’ to 3’)** | | |
| --- | --- | --- | --- |
| *GLI1* | | Forward | CCCAGTACATGCTGGTGGTT |
|  | | Reverse | GCTTTACTGCAGCCCTCGT |
| *GLI2* | | Forward | CCACTCCAGCCAAGTTGGGA |
|  | | Reverse | CTGCTGTCCTCCAAGAGACC |
| *PTCH1* | | Forward | GGCAGCGGTAGTAGTGGTGTTC |
|  | | Reverse | TGTAGCGGGTATTGTCGTGTGTG |
| *HIP1* | | Forward | TGATTTCACAGGCTCAGTGC |
|  | | Reverse | TGGGATGTCTATCCACAGCA |
| *MEF2C* | | Forward | TGTTCCACCTCCCAACTTCG |
|  | | Reverse | ATTGCCATACCCGTTCCCTG |
| *MEF2D* | | Forward | GTGCTGCTCAAGTACACGGA |
|  | | Reverse | CGGGACAGTTGACCCATAGC |
| *GAPDH* | | Forward | AACAGCCTCAAGATCATCAGCAA |
|  | | Reverse | CAGTCTGGGTGGCAGTGAT |
| *18srRNA* | | Forward | CGGTACCACATCCAAGGAA |
|  | | Reverse | GCTGGAATTACCGCGGCT |
| *TBP* | | Forward | CAACAGCCTGCCACCTTAC |
|  | | Reverse | CTGAATAGGCTGTGGGGTC |

**Supplementary Table S3. List and sequences of the primers used for Q-PCR.**
